# A media intervention applying debunking versus non-debunking content to combat vaccine misinformation in elderly in the Netherlands: A digital randomised trial

**DOI:** 10.1016/j.eclinm.2021.100881

**Published:** 2021-05-15

**Authors:** Hamza Yousuf, Sander van der Linden, Luke Bredius, G.A. (Ted) van Essen, Govert Sweep, Zohar Preminger, Eric van Gorp, Erik Scherder, Jagat Narula, Leonard Hofstra

**Affiliations:** a Department of Cardiology, Amsterdam UMC, VU University medical center, De Boelelaan 1117 – 1118, 1081 HV Amsterdam, the Netherlands; b Department of Psychology, School of Biology, University of Cambridge, Cambridge, UK; c Dutch Influenza Foundation, Amersfoort, the Netherlands; d Department of Viroscience, Erasmus MC, Rotterdam, the Netherlands; e Department of Clinical Neuropsychology, VU University, Amsterdam, the Netherlands; f Mount Sinai, St. Luke's Hospital, New York, NY, USA

**Keywords:** Vaccines, Misinformation, Public health, Media psychology, Debunking, Media intervention

## Abstract

**Background:**

As several COVID-19 vaccines are rolled-out globally, it has become important to develop an effective strategy for vaccine acceptance, especially in high-risk groups, such as elderly. Vaccine misconception was declared by WHO as one of the top 10 health issues in 2019. Here we test the effectiveness of applying debunking to combat vaccine misinformation, and reduce vaccine hesitancy.

**Methods:**

Participants were recruited via a daily news show on Dutch Television, targeted to elderly viewers. The study was conducted in 980 elderly citizens during the October 2020 National Influenza Vaccination Campaign. Borrowing from the recent literature in behavioural science and psychology we conducted a two-arm randomized blinded parallel study, in which participants were allocated to exposure to a video containing social norms, vaccine information plus debunking of vaccination myths (intervention group, *n* = 505) or a video only containing vaccine information plus social norm (control group, *n* = 475). Participants who viewed either of the video's and completed both a pre- and post-intervention survey on vaccination trust and knowledge, were included in the analysis. The main outcomes of this study were improvement on vaccine knowledge and awareness.

**Findings:**

Participants were recruited from the 13th of October 2020 till the 16th of October 2020 and could immediately participate in the pre-intervention survey. Subsequently, eligible participants were randomly assigned to an interventional video and the follow-up survey, distributed through email on the 18th of October 2020, and available for participation till the 24th of October 2020. We found that exposure to the video with addition of debunking strategies on top of social norm modelling and information resulted in substantially stronger rejection of vaccination misconceptions, including the belief that: (1) vaccinations can cause Autism Spectrum Disorders; (2) vaccinations weaken the immune system; (3) influenza vaccination would hamper the COVID-19 vaccine efficacy. Additionally, we observed that exposure to debunking in the intervention resulted in enhanced trust in government.

**Interpretation:**

Utilizing debunking in media campaigns on top of vaccine information and social norm modeling is an effective means to combat misinformation and distrust associated with vaccination in elderly, and could help maximize grounds for the acceptance of vaccines, including the COVID-19 vaccines.

**Funding:**

Dutch Influenza Foundation.

**Research in context**

**Evidence before this study**

Vaccination hesitancy is regarded as a top 10 health priority by the WHO. Recent studies in behavioural science suggest that debunking vaccination myths should be an effective method to increase vaccination confidence. In addition, debunking scripts have been developed. Here, we used these latest insights in behavioural science to investigate the impact of debunking, on top of information and social norm modelling, in a randomised video intervention study in the 980 elderly citizens.

**Added value of this study**

The results show that adding debunking scripts on top of vaccine information and social norm modelling resulted in stronger rejection of vaccination myths, and enhanced trust in the COVID-19 mitigation measures by the government. These results suggest that debunking is an effective communication strategy in public health messaging, to enhance vaccination confidence. Based on these results, the intervention video with debunking was used in a widespread campaign via television.

**Implications of all the available evidence**

On the basis of this study and the other existing evidence, the strategy to use evidence based health campaigning using the latest insights in behavioural science, could serve as an effective and relatively low cost method to convey public health messages.

Alt-text: Unlabelled box

## 1 Introduction

At least 3 COVID-19 vaccine candidates have received emergency use authorization (EUA) [1]. A major challenge, however, will be to maximize acceptance of the novel vaccines which have been developed at an unprecedented pace, compared to previous vaccine development timelines. The World Health Organization (WHO) declared vaccine hesitancy as one of the top 10 global health threats in 2019, as they referred to the uprising in anti-vax myths by individuals and bots actively spreading fake news, especially on social media [2,3]. This, in turn, is fuelling distrust in science, governments and health organizations as an *infodemic* [4]. This was amplified during the COVID-19 pandemic, and could pose a challenge to public acceptance of the COVID-19 vaccine, on top of its effects on vaccine hesitancy in general [5]. A striking example is the renewed societal hesitancy against measles vaccination, which has led to an increase of 31% in measles cases reported globally [6]. This calls for effective measures to counteract misinformation and behaviour pertaining to vaccine hesitancy [2].

To enhance public confidence in vaccination, governments worldwide should implement public health strategies to reduce vaccine refusal, as the intention to accept a COVID-19 vaccine is non-optimal and is even decreasing [7,8]. In addition, in as much as a third to half of the population in several countries, such as Germany, France and Japan do not plan to take a COVID-19 vaccination [9]. Optimal vaccination levels are particularly important in high-risk subjects including elderly and those with pre-existing heart or respiratory disease. Furthermore, on top of health determinants of high-risk, social characteristics play an important part in COVID-19 vulnerability. For instance, it has been observed that the African American population has been hit disproportionately by the COVID-19 pandemic, and moreover seem to be at higher risk for vaccine refusal [10]. Social and behavioural science evidence has demonstrated that debunking can effectively mitigate misperceptions including vaccination myths, and therefore, could help to decrease vaccine refusal [11,12]. We have previously demonstrated that evidence-based campaigning and entertainment-based interventions can effectively promote awareness and influence behaviours on a mass scale [[13], [14], [15]]. In the randomized study presented here we aimed to test the hypothesis that a video containing evidence-based content, social norm modelling and debunking strategies should help to improve knowledge and awareness about vaccines in targeted individuals, compared to a video containing information and social norms without debunking strategies. We tested this Public Health communication strategy during the National Influenza Vaccination Campaign for elderly in the Netherlands, in October of 2020.

## 2 Methods

### 2.1 Study design

This study was designed as a randomized parallel-group blinded study, in the Netherlands. A video intervention in 2 different versions was randomly assigned via Qualtrics (digital randomisation) to the participants. A follow up survey was used to measure the outcomes.

This study was reviewed by the Medical Ethical Review Board (METC) of the Amsterdam UMC and VU University Medical Center, and was exempted from the need for an IRB approval. Participants were required to give informed consent for participation by ticking the ‘I have read the information given above and agree to participate in this study’-box in the digital informed consent form that preceded the start of the pre-intervention survey. If participants did not provide informed consent, they were redirected to the end of the survey and excluded for participation.

This study follows the CONSORT 2010 guidelines for reporting randomized trials.

### 2.2 Participants and randomisation

**Fig. 1:**
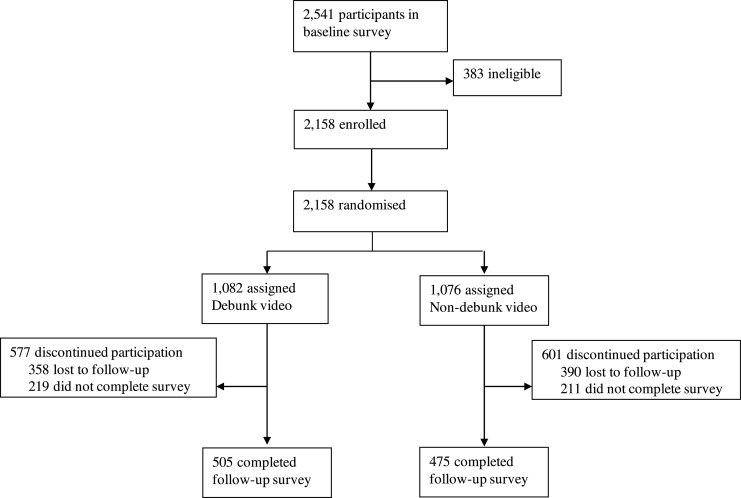
A flow-chart describing the trial profile of the study.

Due to the restricted sampling population from viewers of the television channel and opportunistic participation, we did not pre-determine a sample size. Participants who filled out the diagnostic survey (*n* = 2541), were assessed on eligibility (1. gave informed consent, 2. provided an email address), and *n* = 2158 participants were randomized with an equal allocation ratio (1:1) to a Qualtrics link containing either Video 1 (*n* = 1076) without debunk strategies, or Video 2 (*n* = 1082) with debunk strategies, and a questionnaire that was identical for both conditions (Fig. 1). Of the *n* = 2158 randomized participants who were invited to view an Influenza campaign video and fill the follow-up survey, *n* = 748 participants were lost to follow-up, and *n* = 430 participants did not complete the follow-up survey. This approach yielded *n* = 475 included participants for the non-debunking group, and *n* = 505 included participants for the debunking group that filled all fields of the survey. The study was performed in a blinded fashion, in which participants were not aware of the existence of the different videos. Since the videos were posted on our own YouTube channel, the videos were only visible on invitation, which minimized the possibility of a spill-over effect between groups.

### 2.3 Procedures

#### 2.3.1 Diagnostic survey development

To evaluate vaccine awareness, knowledge, misconceptions and hesitancy in the Netherlands, we developed a survey, to evaluate (A) demographic information (age, gender, migration background, education, annual income, living surrounding, political view, religion, and e-mail (11 questions), (B) governmental trust on influenza vaccination (7 questions, adapted from WHO SAGE) [16], (C) vaccine hesitancy (10 questions, adapted from a governmental trust survey during the H1N1 pandemic) [17], and (D) myths and knowledge about influenza and COVID-19 (6 questions) (Supplemental data).

#### 2.3.2 Distribution of the survey

The diagnostic survey was promoted through a daily talk show with an average audience of 600,000 daily viewers and was made available for participation on the digital platforms of Omroep Max (the TV channel broadcasting the show) on the 13th of October 2020. The target audience of Omroep MAX is within the same age group (60+) as those susceptible for influenza and SARS-CoV-2 infection complications. On an evening talk show a well-known Dutch physician- Ted van Essen, MD, requested viewers to participate in the vaccination survey available on Omroep MAX website.

#### 2.3.3 Formulation of the videos

**Fig. 2:**
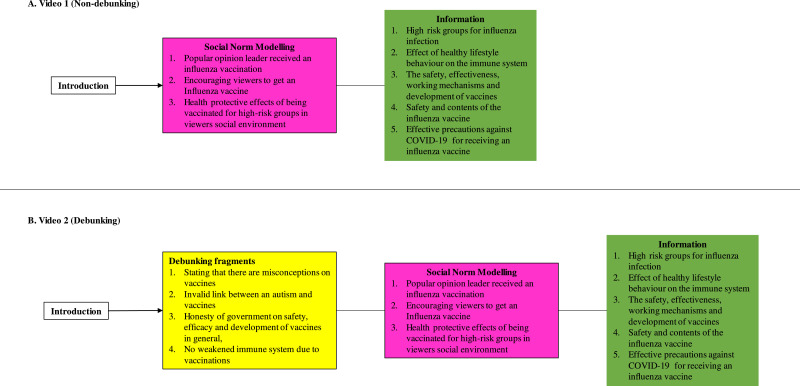
*The general structure of both interventional videos showing which strategies were implemented and which themes were discussed*. The coloured blocks represent Debunking fragments (labelled yellow; only in B. Video 2 (Debunking)), Social Norm Modelling (labelled pink), and Information (labelled green) in the respective contents of either video.

The outcomes of the diagnostic survey were used to design videos aiming at the gaps in understanding and misconceptions surrounding vaccinations (Table 1; description of outcomes in the Results section). In both videos three different TV celebrity scientists (Prof. Erik Scherder, Prof. Dr. Eric van Gorp, Dr. Ted van Essen), the Dutch state secretary of Health, Welfare and Sport (Paul Blokhuis), and Prof. Dr. Leonard Hofstra (professor in Cardiology) were displayed, explaining the different aspects of vaccination, including social norm, information on vaccinations. Furthermore, The control video (Video 1) contained only information on vaccination and social norms (Non-debunking video) (Fig. 2). Video 2 contained all the contents of Video 1, but on top also had several Debunking fragments on vaccination misconceptions. The length of the Non-debunking video (social norms and information only) was 5 min and 11 s, and 6 min and 43 s for the Debunking video (social norms, information and debunking) (Fig. 2). A full time-stamped transcript of both videos can be found in the Supplemental data.

**Table 1:** Demographic characteristics of participants at baseline.

	**Non-debunked (*n*** **=** **475)**		**Debunked (*n*** **=** **505)**		
**Characteristics**	**Mean**	**SD, Range**	**Mean**	**SD, Range**	***P-value***
**Age, y**	69,33	7593, [33 - 94]	69,15	7963, [30 - 95]	0,378
**Gender**	**Frequency**	**Valid (%)**	**Frequency**	**Valid (%)**	
Male	193	40,60%	191	37,80%	0,368
Female	282	59,40%	314	62,20%	
Other	0	0,00%	0	0,00%	
**Migration background**					
Yes	11	2,32%	19	3,80%	0,181
No	460	96,84%	485	96,00%	
Unknown	4	0,84%	1	0,20%	
**Education**					
Elementary school	21	4,43%	18	3,56%	0,227
High school	109	23,00%	93	18,42%	
*Prevocational secondary education*	53	11,18%	56	11,09%	
*Senior general secondary education*	22	4,64%	22	4,36%	
*Preuniversity education*	110	23,21%	104	20,59%	
Secondary vocational education	131	27,64%	161	31,88%	
Higher proffesional education	7	1,48%	13	2,57%	
University education	21	4,43%	38	7,52%	
**Income annual**					
Low income	267	69,20%	283	68,20%	0,766
High income	119	30,80%	132	31,80%	
**Living surrounding**					
City/Urban environment	307	64,80%	332	65,70%	0,715
Rural environment	167	35,20%	173	34,30%	
**Political view**					
Progressive	234	49,30%	247	49,20%	0,985
Conservative	241	50,70%	255	50,80%	
**Religious**					
Yes	175	37,00%	159	31,50%	0,077
No	298	63,00%	345	68,50%

#### 2.3.4 Applied psychological theories

In both videos, we utilized Social norms theory aimed at influencing public opinion through acclaimed experts. Social norms convey information about what others are doing (descriptive norms) as well as prescriptions about what is desirable in society and how people ought to behave (prescriptive norms). In particular, it has been reported that explanation of herd immunity and the social benefits of vaccination can induce prosocial vaccination decisions. On top of that, in the debunking video we also applied key insights from the cognitive psychology literature on how to effectively correct falsehood, by providing (1) social consensus, (2) supporting evidence, (3) consistency, (4) coherence, and (5) credibility [11,12,[18], [19], [20], [21]].

#### 2.3.5 Informational content in both videos

In the videos we provided information on (1) groups at higher risk for an influenza infection and its complications, (2) the effect of healthy lifestyle behaviour on the immune system, (3) the safety, effectiveness, working mechanisms and development of vaccines in general, (4) the safety and contents of the influenza vaccine, and that (5) GP practices have taken effective precautions against COVID-19 to ensure safety of individuals receiving an influenza vaccine.

#### 2.3.6 Content modelling social norms in both videos

We modelled social norms by presenting video graphic material in which the scientific popular opinion leader received an influenza vaccination, as well as by encouraging viewers to let themselves be vaccinated against Influenza for themselves, but also for the health protective effects for people at higher risk for complications during an influenza infection in their social environment (e.g. their parents, grandparents, frail individuals in their social circle).

#### 2.3.7 Debunking content in Video 2

In the video containing debunking fragments (Video 2), we stated in the introduction that there are a lot of misconceptions on vaccines, that we will address these in this video, explain that they were falsely conveyed to the public and provide scientific facts on the actual workings. Subsequently, in Video 2 we debunked various myths that we provide information on in both videos, including (1) the safety, efficacy and development of vaccines in general, (2) the falsely proclaimed link between autism and vaccines, (3) the safety and contents of the influenza vaccine.

#### 2.3.8 Distribution of the intervention and follow-up survey

The interventional videos and the follow-up survey were distributed through email to the participants on the 18th of October 2020, which contained a link to Qualtrics^XM^. When accessing the link, participants were able to view either Video 1 or Video 2, based on the condition they were randomized to. After viewing either one of the videos, participants could directly fill the follow-up survey, which consisted of the same questions as the diagnostic survey excluding demographic information.

### 2.4 Outcomes

#### 2.4.1 *Re*-coding variables and data analysis

Ordinal data was collected through a 4-point Likert scale (for questions 12, 13, 14, 15, 16, 17, 18, 21, 22, 23, 24, 25, 26, 27, 28) and a 5-point Likert scale (for questions, 29, 30, 31, 32, 33, 34). These scales then were converted into numerical values (0, 1, 2 and 3, and 0, 1, 2, 3, and 4, respectively. Although, questions 24, 27, 31, 32, 33 and 34 were reverse coded, due to the nature of the questions.

Question 5, regarding educational level of the respondent, was re-coded into a binary variable, as 0 for Lower Educated (consisting out of *Elementary school, Pre-vocational secondary education*, and *Secondary vocational education*), and 1 for Higher Educated (consisting out of all the remaining levels of education). Question 6, regarding annual income of the respondent, was re-coded into a binary variable, as 0 for Lower Income (below €40.000 annual income), and 1 for Higher Income (same or higher than €40.000 annual income). Question 8, regarding which political party the responded would vote on, was re-coded into a binary variable, as 0 for progressive parties, and 1 for conservative voting preference [22].

### 2.5 Statistical analysis

Nominal and Binary data were analysed with the Chi-Square test to assess differences between both arms of the intervention for demographics, as well as differences between pre-test and post-test outcomes. For all outcomes, an ANCOVA was performed to assess the pre-to-post differences, in which the post-test outcomes were used as dependent variables, the pre-test outcomes were used as covariates, and were compared between the two arms of the study. When the latter was found to be statistically significant (p < .05), linear regression was performed for outcomes that were converted into numerical data, and a logistic regression for binary outcomes, in which was corrected for sex, religion and political views

The result are reported as means, and mean changes (post-pre) for each arm of the intervention, with SDs and 95% CIs. Analysis was conducted using IBM SPSS Statistics for Mac, version 26.0, Armonk, NY, IBM Corp.

### 2.6 Role of the funding source

This study was funded by the Dutch Influenza Foundation. The funder had no role in study design, data collection, data analysis, data interpretation, or writing of the report. Furthermore, the funder had no access to collected data and was not involved in the decision to submit the findings of this study for peer-reviewed publication. All authors had full access to all the data in the study and had final responsibility for the decision to submit for publication.

## 3 Results

The pre-intervention survey was completed by 2541 participants, of which 2158 were eligible for participation and were randomized to either the non-debunking Video 1, which yielded *n* = 475 included participants, or the debunking Video 2, which yielded *n* = 505 included participants (Fig. 1*)*. This study was performed between October 13th and October 24th. All participants completed the post-intervention survey after viewing either video. There was no significant difference in baseline characteristics between those randomly assigned to either the non-debunking or debunking intervention (Table 1). The mean (SD) age of the group assigned to exposure to the Non-debunking video was 69.33 (7.59), and the group assigned to the video containing debunking was 69.15 (7.96) years; 41 and 38 percent were male participants, respectively. In the group assigned to the non-debunking video, 69% participants had a low-income status, 49% had a progressive political view, and 37% reported to adhere to any religion. In the group assigned to the debunking video, 68% of the participants had low-income status, 49% had progressive political views and 32% reported to adhere to any religion.

Through the diagnostic survey we observed that participants that participated in the diagnostic survey (*n* = 2541) generally reported that (1) they could think of a reason to not get vaccinated, (2) they were concerned about side effects of vaccinations, (3) new vaccines carried more risks than older already existing vaccines, (4) they did not know whether getting vaccinated lowered the strength of the immune system, (5) taking an influenza vaccine might affect the effectiveness of a potential COVID-19 vaccine, (6) vaccines can lead to an autism spectrum disorder, (7) an influenza vaccine is not as effective as quitting smoking on reducing the chances of a heart attack during an influenza epidemic, (8) vaccines can protect you against serious illnesses, and (9) vaccines for diseases that no longer occur are not needed. Furthermore, participants reported low trust in the government regarding handling the Influenza virus (i.e., data for participants of the diagnostic survey that were not included in the final analysis is not shown).

**Fig. 3:**
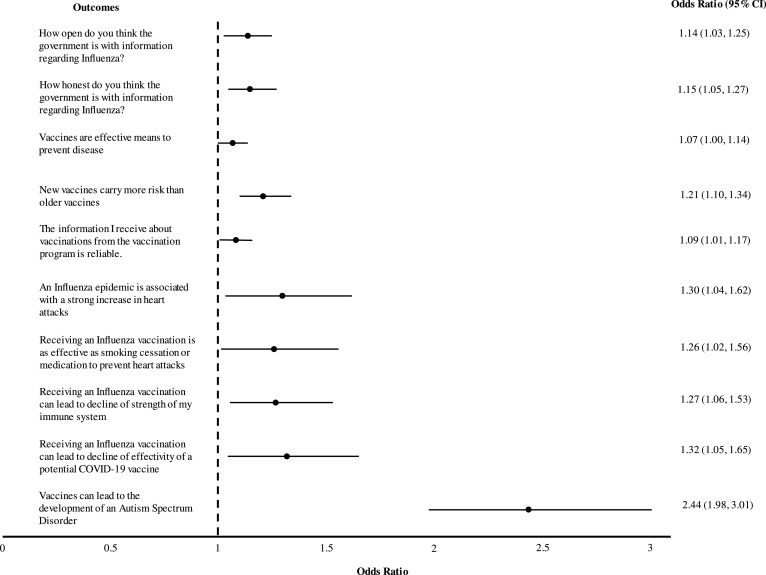
Forest-plot depicting effect of the debunking video as compared to the non-debunking video, for all outcomes that were significantly different between both conditions.

Table 2. summarizes mean outcomes measured in all participants as well as mean change pre and post intervention. Outcomes that were found to be significantly different between groups, underwent regression analysis corrected for sex, religion and political views (Table 3; Fig. 3).

**Table 2:** *Overview of results for all outcomes in both groups***.** Δ The mean difference between post and pre outcomes.

	**Non-Debunked Group *n*** **=** **475**							**Debunked Group *n*** **=** **505**							
**Outcome variable**	**Mean Pre-test**	**SD**	**Mean Post-test**	**SD**	**Δ Post -Pre**	**SD**	**P-value pre-post**	**Mean Pre-test**	**SD**	**Mean Post-test**	**SD**	**Δ Post -Pre**	**SD**	**P-value pre-post**	**P-value pre-post both groups**
How committed do you think the government is to protect you from influenza?	3,36	0,767	3,55	−0,652	0,198	0,658	0,000	3,41	0,712	3,66	0,573	0,254	0,654	0,000	0,185
How much care and concern do you think the government has shown about people who may be affected by a flu outbreak?	3,06	0,847	3,31	0,765	0,251	0,776	0,000	3,03	0,817	3,36	0,753	0,331	0,766	0,000	0,104
How open do you think the government is with information regarding Influenza?	3,04	0,887	3,25	0,848	0,206	0,765	0,000	3,07	0,881	3,4	0,789	0,337	0,765	0,000	0,008
How competent do you think the government is in dealing with influenza	2,91	0,869	3,2	0,823	0,291	0,755	0,000	2,94	0,816	3,29	0,823	0,345	0,761	0,000	0,271
How honest do you think the government is with information regarding Influenza?	2,99	0,896	3,23	0,855	0,242	0,734	0,000	2,98	0,877	3,36	0,808	0,384	0,742	0,000	0,003
To what extent do you believe that the actions of the government in response to influenza is in your personal interest?	3,05	0,906	3,3	0,867	0,257	0,788	0,000	3,07	0,909	3,38	0,827	0,311	0,819	0,000	0,293
To what extent do you think the government will protect you against Influenza?	2,94	0,86	3,17	0,796	0,234	0,719	0,000	2,91	0,838	3,18	0,793	0,267	0,728	0,000	0,467
Do you believe vaccinations can protect you against serious illnesses?	1,9	0,299	1,93	0,262	0,025	0,267	0,000	1,94	0,244	1,95	0,225	0,01	0,256	0,000	0,356
Are there any reasons you can think of why you should not get vaccinated?	1,71	0,455	1,75	0,434	0,04	0,417	0,000	1,68	0,468	1,73	0,444	0,054	0,361	0,000	0,591
Vaccinations are important for my health	3,38	0,723	3,49	0,763	0,118	0,58	0,000	3,38	0,695	3,5	0,655	0,119	0,547	0,000	0,980
Vaccines are effective means to prevent disease	3,23	0,638	3,28	0,629	0,055	0,56	0,000	3,17	0,625	3,3	0,626	0,123	0,52	0,000	0,049
Getting vaccinated is important to the health of others in my community	3,32	0,676	3,45	0,629	0,137	0,555	0,000	3,33	0,672	3,43	0,62	0,097	0,585	0,000	0,275
New vaccines carry more risks than older vaccines	2,48	0,762	2,46	0,795	−0,019	0,754	0,000	2,39	0,761	2,55	0,97	0,166	0,807	0,000	0,000
The information I receive about vaccinations from the vaccination program is reliable.	3,03	0,638	3,13	0,658	0,11	0,564	0,000	3,01	0,642	3,21	0,642	0,202	0,58	0,000	0,012
Getting vaccinations is a good way to protect myself from illnesses.	3,24	0,694	3,34	0,653	0,099	0,541	0,000	3,23	0,662	3,35	0,665	0,121	0,515	0,000	0,522
I am concerned about the serious adverse effects of vaccinations.	2,85	0,806	2,92	0,83	0,07	0,736	0,000	2,83	0,773	2,97	0,811	0,139	0,719	0,000	0,137
I do need vaccinations for diseases that no longer occur.	2,46	0,845	2,65	0,805	0,196	0,754	0,000	2,47	0,787	2,63	0,821	0,162	0,736	0,000	0,478
‘An influenza epidemic (flu wave) is associated with a strong increase in heart attacks’	1,92	1228	2,9	1603	0,983	1729	0,000	1,89	1227	3,11	1,56	1226	1756	0,000	0,030
‘Receiving an Influenza Vaccination (Flu shot) is as effective as smoking cessation or medication to prevent heart attacks’	2,19	1264	2,56	1462	0,371	1636	0,000	2,05	1,22	2,65	1526	0,6	1746	0,000	0,035
‘Flu vaccination can actually lead to flu’	3,49	1366	3,67	1322	0,173	1381	0,000	3,37	1376	3,63	1,36	0,254	1361	0,000	0,356
‘Receiving an Influenza Vaccination (Flu shot) can lead to decline of strength of my immune system’	3,36	1449	3,56	1356	0,2	1431	0,000	3,14	1505	3,59	1393	0,452	1522	0,000	0,008
‘Receiving an Influenza Vaccination (Flu shot) can lead to decline of effectivity of a potential COVID-19 vaccine’	2,75	1674	2,83	1716	0,082	1843	0,000	2,65	1698	3,03	1715	0,382	1775	0,000	0,010
‘Vaccines can lead to the development of an Autism Spectrum Disorder’	3,14	1738	3,23	1765	0,089	1565	0,000	3,06	1751	4,06	1446	0,994	1762	0,000	0,000

**Table 3:** **Overview of regression analysis results of the debunking-group compared with the non-debunking group**. Regression results are shown with and without correction for Sex, Religion and Political view for each significantly different outcome between both conditions.

**Variable**	**R2**	Adjusted R2*	**OR (95% CI)**	**Lower Bound**	**Upper Bound**	Adjusted OR (95% CI)*	**Lower Bound**	**Upper Bound**	***P-value***
**How open do you think the government is with information regarding Influenza?**	0,007	0,01							
Condition (Non-Debunked > Debunked)			1,14	1,03	1,25	1,14	1,03	1,25	0,008
Sex (Female > Male)						0,99	0,89	1,09	0,820
Religion (Non > Religious)						1,03	0,93	1,15	0,533
Political View (Progressive > Conservative)						0,92	0,83	1,02	0,107
**How honest do you think the government is with information regarding Influenza?**	0,009	0,013							
Condition (Non-Debunked > Debunked)			1,15	1,05	1,27	1,15	1,05	1,27	0,003
Sex (Female > Male)						0,95	0,87	1,05	0,335
Religion (Non > Religious)						1,04	0,94	1,15	0,436
Political View (Progressive > Conservative)						0,92	0,84	1,02	0,107
**Vaccines are effective means to prevent disease**	0,004	0,007							
Condition (Non-Debunked > Debunked)			1,07	1,00	1,15	1,07	1,00	1,14	0,056
Sex (Female > Male)						0,96	0,90	1,03	0,269
Religion (Non > Religious)						0,97	0,90	1,05	0,448
Political View (Progressive > Conservative)						0,98	0,91	1,05	0,529
**New vaccines carry more risks than older vaccines**	0,014	0,019							
Condition (Non-Debunked > Debunked)			1,21	1,09	1,33	1,21	1,10	1,34	0,000
Sex (Female > Male)						0,96	0,87	1,07	0,459
Religion (Non > Religious)						1,12	1,00	1,24	0,044
Political View (Progressive > Conservative)						0,96	0,87	1,06	0,409
**The information I receive about vaccinations from the vaccination program is reliable**	0,006	0,012							
Condition (Non-Debunked > Debunked)			1,10	1,02	1,18	1,09	1,01	1,17	0,019
Sex (Female > Male)						1,09	1,02	1,18	0,018
Religion (Non > Religious)						1,02	0,94	1,10	0,713
Political View (Progressive > Conservative)						1,01	0,94	1,09	0,769
**‘An influenza epidemic (flu wave) is associated with a strong increase in heart attacks’**	0,005	0,011							
Condition (Non-Debunked > Debunked)			1,29	1,03	1,60	1,30	1,04	1,62	0,019
Sex (Female > Male)						0,94	0,75	1,18	0,608
Religion (Non > Religious)						1,31	1,03	1,67	0,026
Political View (Progressive > Conservative)						0,89	0,71	1,11	0,299
									
**‘Receiving an Influenza Vaccination (Flu shot) is as effective as smoking cessation or medication to prevent heart attacks’**	0,004	0,008							
Condition (Non-Debunked > Debunked)			1,25	1,01	1,55	1,26	1,02	1,56	0,034
Sex (Female > Male)						1,00	0,80	1,24	0,964
Religion (Non > Religious)						1,17	0,93	1,48	0,178
Political View (Progressive > Conservative)						1,10	0,88	1,37	0,407
**‘Receiving an Influenza Vaccination (Flu shot) can lead to decline of strength of my immune system’**	0,006	0,009							
Condition (Non-Debunked > Debunked)			1,26	1,05	1,52	1,27	1,06	1,53	0,011
Sex (Female > Male)						1,18	0,97	1,43	0,090
Religion (Non > Religious)						1,04	0,85	1,27	0,737
Political View (Progressive > Conservative)						0,96	0,79	1,16	0,667
**‘Receiving an Influenza Vaccination (Flu shot) can lead to decline of effectivity of a potential COVID-19 vaccine’**	0,006	0,008							
Condition (Non-Debunked > Debunked)			1,32	1,05	1,65	1,32	1,05	1,65	0,018
Sex (Female > Male)						1,14	0,90	1,44	0,274
Religion (Non > Religious)						0,93	0,72	1,19	0,562
Political View (Progressive > Conservative)						1,14	0,90	1,44	0,289
									
**‘Vaccines can lead to the development of an Autism Spectrum Disorder’**	0,067	0,076							
Condition (Non-Debunked > Debunked)			2,45	1,98	3,02	2,44	1,98	3,01	0,000
Sex (Female > Male)						1,29	1,04	1,59	0,022
Religion (Non > Religious)						0,80	0,64	1,01	0,059
Political View (Progressive > Conservative)						0,94	0,76	1,17	0,578

The results show that participants exposed to the debunking video were significantly more likely to reject the myth that vaccines could lead to the development of an Autism Spectrum Disorder (odds ratio for disagreement, 2.44 [95% CI, 1.98–3.01]), compared to participants exposed to the non-debunking control video.

Participants exposed to the debunking intervention were significantly more likely to agree that an influenza epidemic is associated with a strong increase in heart attacks (OR 1.30 [95% CI, 1.04–1.62]), and that vaccination for influenza is as effective as smoking cessation or medication to prevent heart attacks (OR 1.26 [95% CI, 1.02–1.56]), respectively, compared to participants exposed to the non-debunking control video.

Participants exposed to the debunking intervention were significantly more likely to reject that an influenza vaccination could result in a weakened immune system (OR 1.27 [95% CI, 1.06–1.53]), and to reject that receiving influenza vaccination could lead to lower efficacy of COVID-19 vaccine (OR, 1.32 [95% CI, 1.05–1.65]), respectively, compared to participants exposed to the non-debunking control video.

Participants exposed to the debunking intervention were significantly more likely to have a more favourable view about how open the government was (OR 1.14 [95% CI, 1.03–1.25]), how honest the government was with information regarding influenza virus (OR 1.15 [95% CI, 1.05–1.27]), and the reliability of information on vaccines provided by the national vaccination program (OR 1.09 [95% CI, 1.01–1.17]), respectively, compared to participants exposed to the non-debunking control video.

Finally, participants exposed to the debunking intervention were significantly more likely to agree that new vaccines do not carry greater risks than the older vaccines (OR 1.21 [95% CI, 1.10–1.34]). However, exposure to the debunking video was not associated with a mean change in the belief concerning vaccination effectiveness on preventing disease (OR 1.07 [95% CI, 1.00–1.14]; *P* = 0.056), respectively, compared to participants exposed to the non-debunking control video.

## 4 Discussion

The current COVID-19 pandemic has resulted in a renewed public interest in vaccinations as an effective tool for prevention, but has also fuelled intense anti-vax sentiments in significant numbers of the population, wordlwide [23]. The latter may pose a threat to the efforts to curb COVID-19 pandemic. However, an increasing demand for other vaccinations, such as the influenza vaccine, has been observed [24]. The efficacy of influenza vaccination in a meta-analysis of high risk individuals including patients with underlying cardiovascular disease showed a 30–40% reduction in cardiovascular events [25]. This preventive effect of Influenza vaccination in high risk groups is as good as quitting smoking or even the prescription of statins. Despite these convincing benefits, acceptance of influenza vaccination in high risk groups is only about 60% [26]. This suggests that we could still accrue a substantial health benefit with increased vaccination coverage even in a known disease, with a vaccine which is known to be safe and effective, such as influenza. Even more, given that vaccine refusal is even prominent in generally known vaccines, most governments and health institutions foresee that a major effort must be invested for acceptance of newer vaccines, such as for COVID-19.

Here, we applied the latest insights in behavioural science to decrease the risk of vaccination hesitancy. Recent work on debunking of misinformation has demonstrated that its success relies in part on the credibility of the communicator [11,12,18]. For this reason, we utilized well respected scientists and doctors to provide not only the social norm and information, but also the debunking statements in the intervention videos. In addition, we used the recommended debunking communication strategy by first providing the truth, followed by warning about the myth, and reiterating the truth at end [19,20]. The data presented here show that the debunking strategies were effective in increasing knowledge and awareness surrounding vaccinations, combatting vaccine misinformation, and enhanced the trust in governmental institutions. The use of randomized trials as a tool to investigate the effect of campaign-like interventions is unusual, but is advocated by internationally renowned behavioural scientists [27]. Our results also show that the debunking strategy, on top of social norm and vaccine information, helped reject misconception that vaccination caused autism, weakened the immune system, and that the influenza vaccination adversely affected the efficacy of COVID-19 vaccination. Intriguingly, exposure to the debunking video also improved the knowledge pertaining to the effect of influenza vaccination in preventing cardiovascular events more. These findings suggest that juxta-positioning the debunking of myths with information on vaccination efficacy and social norms modelling might benefit in retaining the facts about vaccination, and that the familiarity with authentic scientific information could help change beliefs in targeted participants. We would recommend further research to explore the effects of debunking vaccine misinformation on the willingness to get vaccinated. Of importance is the fact that 69% (i.e., only shown per condition) of the participants were classified as having a low income, a group known to be at more risk to be hesitant to vaccination. The data presented here show that also in the lower social class, such a media intervention is effective. The strategy of using a diagnostic survey first, to uncover the most prominent gaps in knowledge and behaviour in the target audience, and use this insight for the design of the intervention, increases the chance of creating an effective media campaign. By using existing media as carriers for the campaign ensures that it can be done at a low cost.

We believe that governments around the globe could utilize this so-called Evidence Based Campaigning strategy to maximize grounds for vaccine acceptance, not only during the current COVID-19 pandemic but also for existing vaccinations, such as against measles and influenza. In addition, the strategy could also be applicable to other prominent public health issues.

Our study was conducted during the upslope of the second wave of COVID-19 in the Netherlands. Therefore, the data may not be entirely representative for communications outside a pandemic. However, it has been previously shown that debunking is an effective strategy to combat misinformation in general. In addition, the distribution of the survey and intervention were done by a TV channel catering to older viewers, which is indicated by the high median age of 61 years of channel viewers. Nevertheless, it was anticipated that the viewers of the TV channel provided a good representation of elderly in the Netherlands, since it is the most popular medium for Dutch elderly. Requesting online participation through email may have resulted in bias, due to differences in tech savviness of elderly and tendency to respond. However, internet access in the Netherlands remains one of the highest in the world (98% of all households). In addition, all outcomes are self-reported by the participant, which could have resulted in bias related to social desirability of answers. We minimized the possibility of recall bias, since participants were prompted to fill the survey instantly after viewing either Video 1 or Video 2, based on the condition they were randomized to. There was a loss of participants between the diagnostic survey and the post-intervention survey. However, participants were randomized after full completion of the diagnostic survey, and being found eligible for participation (1. provided informed consent; 2. provided a valid email address). The probability that a participant was lost to follow up after this step is assumed to be similar in both arms of the study. The impact of debunking may not be generalizable to other age groups. However, the age group above 60 is particularly vulnerable to the complications of COVID-19, and therefore the data presented here are still relevant. Finally, due to the nature of the study we could only measure short term effects of the video intervention. Therefore, the long term effects of the intervention are not clear, as effects of the intervention might be impacted due to exposure to the vast amount of misinformation disseminated nowadays. We would recommend to further research the sustained effects of such interventions, as well as, the effects of repeated exposure to content applying debunking to combat misinformation.

Due to the execution of this study in the Netherlands, the generalizability of the study results might be bound to Western or high-income and high-to-middle income countries. Nonetheless, vaccine hesitancy is labelled as one of the top 10 global health threats by the WHO, and is largely fuelled by the uprise in vaccine misinformation. We believe that utilizing the approach of this study, by identifying knowledge and behaviour gaps, and tackling misinformation through personalized content, applying debunking, social norm modelling, and accurate vaccine information, remains a powerful broadly applicable concept [3].

In conclusion, we demonstrated that adding debunking to a campaign video, on top of vaccination information and social norm modelling, increased its effectiveness in combating vaccination myths, resulting in higher rejection of misinformation. We hope the results of our work will encourage policy makers to employ debunking in their public health campaigns, including social media campaigns, to positively influence the spectrum of opinions for developing public confidence in vaccinations, including the COVID-19 vaccine.

## Funding

Dutch Influenza Foundation

## Data sharing agreement

The authors agree to make the anonymized data available upon reasonable request to the corresponding/first author.

## Contributors

Dr Yousuf was responsible for literature search, figures, study design, data collection, intervention design, data analysis, data interpretation and writing; Dr van der Linden was responsible for literature search, study design, data analysis, intervention design, data interpretation and writing; Mr Bredius was responsible for literature search, figures, study design, intervention design, data collection, data analysis, data interpretation and writing; Dr van Essen was responsible for literature search and study design; Mr Sweep was responsible for writing and intervention design; Ms Preminger was responsible for literature search and data analysis; Dr van Gorp was responsible for literature search, intervention design, data interpretation and writing; Dr Scherder was responsible for literature search, intervention design, data interpretation and writing; Dr Narula was responsible for data interpretation and writing; Dr Hofstra was responsible for literature search, figures, study design, data collection, intervention design, data analysis, data interpretation and writing

## Declaration of Competing Interest

Dr. Hofstra and Dr Yousuf report grants from Dutch Influenza Foundation, during the conduct of the study. No other authors have any competing interests to report.

## Appendix Supplementary materials

Image, application 1

Image, application 2

Image, application 3
