# Supplementary material for: A media intervention applying debunking versus non-debunking content to combat vaccine misinformation in elderly in the Netherlands: A digital randomised trial
Source: eClinicalMedicine. 2021 May 15;35:100881. doi: 10.1016/j.eclinm.2021.100881 (PMC8176124; doi:10.1016/j.eclinm.2021.100881)
Supplement: Supplementary file 1 [file mmc1.docx]

**Supplemental file 1**

**Study survey**

**A. Demographics**

1. What is your age?

____________ (000-999)

2. What is your gender?

Male  (2)

Female  (1)

3. Do you or your family have a migration background?

Yes   (1)

No  (2)

Unknown (3)

4. What is your ethnicity? Select all countries that apply to you.

Afghanistan  (1)

Albania  (2)

Algeria  (3)

Andorra  (4)

Angola  (5)

Antigua and Barbuda  (6)

Argentina  (7)

Armenia  (8)

Australia  (9)

Austria  (10)

Azerbaijan  (11)

Bahamas  (12)

Bahrain  (13)

Bangladesh  (14)

Barbados  (15)

Belarus  (16)

Belgium  (17)

Belize  (18)

Benin  (19)

Bhutan  (20)

Bolivia  (21)

Bosnia and Herzegovina  (22)

Botswana  (23)

Brazil  (24)

Brunei Darussalam  (25)

Bulgaria  (26)

Burkina Faso  (27)

Burundi  (28)

Cambodia  (29)

Cameroon  (30)

Canada  (31)

Cape Verde  (32)

Central African Republic  (33)

Chad  (34)

Chile  (35)

China  (36)

Colombia  (37)

Comoros  (38)

Congo, Republic of the...  (39)

Costa Rica  (40)

Côte d'Ivoire  (41)

Croatia  (42)

Cuba  (43)

Cyprus  (44)

Czech Republic  (45)

Democratic People's Republic of Korea (46)

Democratic Republic of the Congo (47)

Denmark  (48)

Djibouti  (49)

Dominica  (50)

Dominican Republic  (51)

Ecuador  (52)

Egypt  (53)

El Salvador  (54)

Equatorial Guinea  (55)

Eritrea  (56)

Estonia  (57)

Ethiopia  (58)

Fiji  (59)

Finland  (60)

France  (61)

Gabon  (62)

Gambia  (63)

Georgia  (64)

Germany  (65)

Ghana  (66)

Greece  (67)

Grenada  (68)

Guatemala  (69)

Guinea  (70)

Guinea-Bissau  (71)

Guyana  (72)

Haiti  (73)

Honduras  (74)

Hong Kong (S.A.R.)  (75)

Hungary  (76)

Iceland  (77)

India  (78)

Indonesia  (79)

Iran, Islamic Republic of... (80)

Iraq (81)

Ireland  (82)

Israel  (83)

Jamaica  (84)

Japan  (85)

Jordan  (86)

Kazakhstan  (87)

Kenya  (88)

Kiribati  (89)

Kurdistan  (90)

Kuwait  (91)

Kyrgyzstan  (92)

Lao People's Democratic Republic (93)

Latvia  (94)

Lebanon  (95)

Lesotho  (96)

Liberia  (97)

Libyan Arab Jamahiriya  (98)

Liechtenstein  (99)

Lithuania  (100)

Luxembourg  (101)

Madagascar  (102)

Malawi  (103)

Malaysia  (104)

Maldives  (105)

Mali  (106)

Malta  (107)

Marshall Islands  (108)

Mauritania  (109)

Mauritius  (110)

Mexico  (111)

Micronesia, Federated States of... (112)

Monaco  (113)

Mongolia  (114)

Montenegro  (115)

Morocco  (116)

Mozambique  (117)

Myanmar  (118)

Namibia  (119)

Nauru  (120)

Nepal  (121)

Netherlands  (122)

New Zealand  (123)

Nicaragua  (124)

Niger  (125)

Nigeria  (126)

North Korea  (127)

Norway  (128)

Oman  (129)

Pakistan  (130)

Palau  (131)

Palestine  (132)

Panama  (133)

Papua New Guinea  (134)

Paraguay  (135)

Peru  (136)

Philippines  (137)

Poland  (138)

Portugal  (139)

Qatar  (140)

Republic of Korea  (141)

Republic of Moldova  (142)

Romania  (143)

Russian Federation  (144)

Rwanda  (145)

Saint Kitts and Nevis  (146)

Saint Lucia  (147)

Saint Vincent and the Grenadines (148)

Samoa  (149)

San Marino  (150)

Sao Tome and Principe  (151)

Saudi Arabia  (152)

Senegal  (153)

Serbia  (154)

Seychelles  (155)

Sierra Leone  (156)

Singapore  (157)

Slovakia  (158)

Slovenia  (159)

Solomon Islands  (160)

Somalia  (161)

South Africa  (162)

South Korea  (163)

Spain  (164)

Sri Lanka  (165)

Sudan  (166)

Suriname  (167)

Swaziland  (168)

Sweden  (169)

Switzerland  (170)

Syrian Arab Republic  (171)

Tajikistan  (172)

Thailand  (173)

The former Yugoslav Republic of Macedonia  (174)

Timor-Leste  (175)

Togo  (176)

Tonga  (177)

Trinidad and Tobago  (178)

Tunisia  (179)

Turkey  (180)

Turkmenistan  (181)

Tuvalu  (182)

Uganda  (183)

Ukraine  (184)

United Arab Emirates  (185)

United Kingdom of Great Britain and Northern Ireland (186)

United Republic of Tanzania (187)

United States of America  (188)

Uruguay  (189)

Uzbekistan  (190)

Vanuatu  (191)

Venezuela, Bolivarian Republic of... (192)

Viet Nam  (193)

Yemen  (194)

Zambia  (195)

Zimbabwe  (196)

5. What is the highest level of school you have completed or the highest degree you have obtained?

Elementary school  (1)

Pre-vocational secondary education  (2)

Senior general secondary education  (3)

Pre-university education  (4)

Secondary vocational education   (5)

Higher professional education  (6)

University education  (7)

6. What is your gross annual income?

€19.999 or lower  (1)

€20.000 – €39.999  (2)

€40.000 – €59.999  (3)

€60.000 – €79.999  (4)

€80.000 – €99.999  (5)

€100.000 or higher  (6)

I'd rather not say  (7)

7. Where do you live?

City (or suburban)  (1)

Rural area  (2)

8. Please indicate which political party you are currently most likely to vote for in the national elections?

VVD  (1)

PvdA  (2)

PVV  (3)

SP  (4)

CDA  (5)

D66 (6)

ChristenUnie  (7)

GroenLinks  (8)

SGP (9)

Partij voor de Dieren  (10)

50PLUS  (11)

Ondernemerspartij  (12)

VNL Voor Nederland  (13)

DENK (14)

Nieuwe Wegen  (15)

Forum voor Democratie  (16)

De BurgerBeweging  (17)

Vrijzinnige Partij  (18)

GeenPeil  (19)

Piratenpartij  (20)

Bij1 (21)

Niet Stemmers  (22)

Libertarische Partij  (23)

Lokaal in de Kamer  (24)

JEZUS LEEFT  (25)

StemNL  (26)

Mens en Spirit  (27)

Basisinkomen Partij  (28)

V-R (Vrede & Recht))  (29)

Vrije Democratische Partij  (30)

9. Are you religious?

Yes  (2)

No  (1)

10. Which religion do you adhere to?

Christianity  (1)

Islam  (2)

Judaism  (3)

Hinduism (4)

Buddhism  (5)

Otherwise, namely....  (6)

11. What is your email address?

________________________________________________

**B. Governmental Trust**

12. How committed do you think the government is to protect you from influenza?

Absolutely not  (1)

Somewhat no  (2)

Somewhat yes  (3)

Absolutely  (4)

13. How much care and concern do you think the government has shown about people who may be affected by a flu outbreak?

Absolutely not  (1)

Somewhat no  (2)

Somewhat yes  (3)

Absolutely  (4)

14. How open do you think the government is with information regarding Influenza?

Absolutely not  (1)

Somewhat no  (2)

Somewhat yes  (3)

Absolutely  (4)

15. How competent do you think the government is in dealing with influenza

Absolutely not  (1)

Somewhat no  (2)

Somewhat yes  (3)

Absolutely  (4)

16. How honest do you think the government is with information regarding Influenza?

Absolutely not  (1)

Somewhat no  (2)

Somewhat yes  (3)

Absolutely  (4)

17. To what extent do you believe that the actions of the government in response to influenza is in your personal interest?

Absolutely not  (1)

Somewhat no  (2)

Somewhat yes  (3)

Absolutely  (4)

18. To what extent do you think the government will protect you against Influenza?

 Absolutely not  (1)

 Somewhat no  (2)

 Somewhat yes  (3)

 Absolutely  (4)

**C. Vaccine Hesitancy**

19. Do you believe vaccinations can protect you against serious illnesses?

Yes  (2)

No  (1)

20. Are there any reasons you can think of why you should not get vaccinated?

 Yes (1)

No  (2)

21. Vaccinations are important for my health

Strongly disagree  (1)

Disagree  (2)

Agree  (3)

Strongly agree  (4)

22. Vaccines are effective means to prevent disease

Strongly disagree  (1)

Disagree  (2)

Agree  (3)

Strongly agree  (4)

23. Getting vaccinated is important to the health of others in my community

Strongly disagree  (1)

Disagree  (2)

Agree  (3)

Strongly agree  (4)

24. New vaccines carry more risks than older vaccines

Strongly disagree  (1)

Disagree  (2)

Agree  (3)

Strongly agree  (4)

25. The information I receive about vaccinations from the vaccination program is reliable.

Strongly disagree  (1)

Disagree  (2)

Agree  (3)

Strongly agree  (4)

26. Getting vaccinated is a good way to protect myself from illnesses.

Strongly disagree  (1)

Disagree  (2)

Agree  (3)

Strongly agree  (4)

27. I am concerned about the serious adverse effects of vaccinations.

Strongly disagree  (1)

Disagree  (2)

Agree  (3)

Strongly agree  (4)

28. I do need vaccinations for diseases that no longer occur.

Strongly disagree  (1)

Disagree  (2)

Agree  (3)

Strongly agree  (4)

**D. Knowledge Questions**

29. ‘An influenza epidemic (flu wave) is associated with a strong increase in heart attacks’

Strongly disagree  (1)

Disagree  (2)

Undecided  (3)

Agree  (4)

Strongly agree  (5)

30. ‘Receiving an Influenza Vaccination (Flu shot) is as effective as smoking cessation or medication to prevent heart attacks’

Strongly disagree  (1)

Disagree  (2)

Undecided  (3)

Agree  (4)

Strongly agree  (5)

31. ‘Flu vaccination can actually lead to flu’

Strongly disagree  (1)

Disagree  (2)

Undecided  (3)

Agree  (4)

Strongly agree  (5)

32. ‘Receiving an Influenza Vaccination (Flu shot) can lead to decline of strength of my immune system’

Strongly disagree  (1)

Disagree  (2)

Undecided  (3)

Agree  (4)

Strongly agree  (5)

33. ‘Receiving an Influenza Vaccination (Flu shot) can lead to decline of effectivity of a potential COVID-19 vaccine’

Strongly disagree  (1)

Disagree  (2)

Undecided  (3)

Agree  (4)

Strongly agree  (5)

34. ‘Vaccines can lead to the development of an Autism Spectrum Disorder’

Strongly disagree  (1)

Disagree  (2)

Undecided  (3)

Agree  (4)

Strongly agree  (5)
