# Supplementary material for: A media intervention applying debunking versus non-debunking content to combat vaccine misinformation in elderly in the Netherlands: A digital randomised trial
Source: eClinicalMedicine. 2021 May 15;35:100881. doi: 10.1016/j.eclinm.2021.100881 (PMC8176124; doi:10.1016/j.eclinm.2021.100881)
Supplement: Supplementary file 2 [file mmc2.docx]

**Supplemental file 2

Transcript of Video 1 (Non-debunking)**

***Prof. Erik Scherder:***

00:00

The world has been startled (unsettled)  by the corona virus in the past year.

00:04

It impacts everyone in our society, your parents, your children, your grandparents, your partner, friends, but also yourself.

00:12

There is [civil] unrest and sometimes even suspicion about vaccinations,

00:18

Whereas these vaccinations should protect us from these types of viruses.

00:19

That is why right now it is very important that we together resist [against vaccination hesitancy]

00:26

The first type of vaccine we know of was already developed in 1796.

00:28

The vaccine was an weakened form of the smallpox virus,

00:34

that occured in cows, but actually protected us against the virus

00:36

Today we are going to learn more about what vaccines are, their effects and

00:41

how they protect us from disease.

00:46

[Music]

***Dr. Ted van Essen:***

00:56

We know that the effectiveness of the immune system [of the body] decreases as you get older.

1:01

Your immune system ages in the same way, we call it immunosenescence.

***Secretary of state Paul Blokhuis:***

1:03

Every year it is important that people participate in [taking] the flu vaccination.

1:07

But this year all the more because  we still are in the corona pandemic.

1:12

The second wave is in full swing and by getting the flu vaccine, which is available for free,

1:18

you become more resilient, you are less susceptible to the flu, and you feel better.

1:25

And that is particularly important nowadays, especially for the vulnerable groups.

***Dr. Ted van Essen:***

1:29

People over 60 are more at risk for complications [from the flu], one could think about..

1:33

pneumonia, heart disease or strokes, for example.

1:37

The same goes for people with diabetes, COPD, chronic lung disease, they are also much more susceptible.

1:44

If you've already have had a myocardial infarction, or have any other heart condition, you are also

1:48

much more susceptible to the complications of influenza.

***Prof. Dr. Leonard Hofstra:***

1:49

Flu is actually much more dangerous than we think.

1:53

During a flu wave, the chance is six times higher that you can have a heart attack

gets.

1:57

This is life-threatening for people who have underlying heart disease.

2:01

That's why it's so important to get vaccinated.

***Dr. Ted van Essen:***

2:04

[In terms of death from the flu] there are differences between the years,

2:07

In 2018 there were nearly 10,000 people who died from it in the Netherlands

2:10

This year it was a minor epidemic,

2:13

just before the coronavirus entered the country.

2:14

There were 400 extra deaths [from the flu].

2:17

On average over the past ten years  we observed 2900 extra deaths per year in the Netherlands

2:22
Which makes the flu a really serious infectious disease.

2:25

If you consider all infectious diseases, influenza is the most common and the most deadly.

***Secretary of state Paul Blokhuis:***

2:31

The flu vaccine has been thoroughly reviewed and we are very transparent about what's in there,

2:38

also about any side effects.

***Prof. Dr. Eric van Gorp:***

2:41

The flu vaccine is effective and safe, because vaccines that come on the market in the Netherlands

2:46

have been extensively tested, in particular for safety. But of course also on effectiveness,

2:52

so you can assume that Influenza vaccinations are safe

***Dr. Ted van Essen:***

2:54

[Side effects] are kept by LAREB in the Netherlands. There you can report side effects,

2:59

and based on this feedback an annual report is issued.

3:02

All you will notice from the flu shot is that you get some pain in the arm where you got it.

3:06

But that's not a side effect, that's where the vaccine starts to work.

***Prof. Erik Scherder:***

3:11

In addition to taking the vaccines, people can take care of

3:13

having an active lifestyle. That actually means they sleep well,

3:18

and also exercise enough during the day.

3:21

The two are closely related. By exercising and sleeping well, you also strengthen your immune system.

***Dr. Ted van Essen:***

3:26

A vaccine contains small pieces of the virus that are dead.

3:32

That's why, if you get the vaccination you cannot get the flu.

3:36

Because the virus in it no longer works.

3:39

The body will make antibodies against those small pieces of the virus.

3:44
If you encounter the actual flu, the body recognizes the virus immediately, and starts using antibodies [produced due to the vaccine] to fight it.

3:54

Thus you prevent serious disease.

***Prof. Dr. Eric van Gorp:***

3:56

A vaccine is considered unsafe if there are [serious] side effects.

4:00

A vaccine in general can cause side effects, mild side effects because your immune system is activated.

4:06

These are expected effects and side effects.

4:10

If unexpected or serious side effects occur,

4:16

it means the end of that vaccine.

***Secretary of state Paul Blokhuis:***

4:17

I would say to the people who get an invitation from their GP to get the flu shot, that the GPs are doing their very best to get people vaccinated,

4:26

make use of it, because it is completely safe.

4:29

I can imagine people are scared.

4:31

That they might think “will it be crowded while I am waiting for a flu shot?”, but this is absolutely not the case.

4:37

The GPs have organized it well, so no fear while getting the vaccine.

***Prof. Erik Scherder:***

4:43

I also take a flu vaccine myself, because I want to protect myself against the effects of the flu,

4:49

and i also want to protect others, by making the chance of getting sick as small as possible.

***Prof. Dr. Leonard Hofstra:***

4:54

so get vaccinated.

4:56

You do this not only for yourself, but also for your loved ones.
 **Transcript of Video 2 (Debunking)**

***Prof. Erik Scherder:***

00:00

The world has been startled (unsettled)  by the corona virus in the past year.

00:04

It impacts everyone in our society, your parents, your children, your grandparents, your partner, friends, but also yourself.

00:12

There is [civil] unrest and sometimes even suspicion about vaccinations,

00:18

Whereas these vaccinations should protect us from these types of viruses.

00:19

That is why right now it is very important that we together resist [against vaccination hesitancy]

00:26

The first type of vaccine we know of was already developed in 1796.

00:28

The vaccine was an weakened form of the smallpox virus,

00:34

that occured in cows, but actually protected us against the virus

00:37

At the time some people even thought

00:39

That they would get cow horns from the smallpox vaccine.

00:43

Today we are going to learn more about what vaccines are, their effects and

00:48

how they protect us from disease.

00:50

There are many misconceptions surrounding vaccinations.

00:54

It is important to identify these inaccuracies and tell the facts about them.

00:59

[Music]

***Dr. Ted van Essen:***

1:10

We know that the effectiveness of the immune system [of the body] decreases as you get older.

01:15

Your immune system ages in the same way, we call it immunosenescence.

***Secretary of state Paul Blokhuis:***

1:18

Every year it is important that people participate in [taking] the flu vaccination.

1:22

But this year all the more because  we still are in the corona pandemic.

1:27

The second wave is in full swing and by getting the flu vaccine, which is available for free,

1:35

you become more resilient, you are less susceptible to the flu, and you feel better.

1:39

And that is particularly important nowadays, especially for the vulnerable groups.

***Dr. Ted van Essen:***

1:44

People over 60 are more at risk for complications [from the flu], one could think about..

1:47

pneumonia, heart disease or strokes, for example.

1:52

The same goes for people with diabetes, COPD, chronic lung disease, they are also much more susceptible.

1:59

If you've already have had a myocardial infarction, or have any other heart condition, you are also

2:02

much more susceptible to the complications of influenza.

***Prof. Dr. Leonard Hofstra:***

2:02

Flu is actually much more dangerous than we think.

2:06

During a flu wave, the chance is six times higher that you can have a heart attack

gets.

2:13

This is life-threatening for people who have underlying heart disease.

2:16

That's why it's so important to get vaccinated.

***Dr. Ted van Essen:***

2:19

[In terms of death from the flu] there are differences between the years,

2:21

In 2018 there were nearly 10,000 people who died from it in the Netherlands

2:24

This year it was a minor epidemic,

2:27

just before the coronavirus entered the country.

2:29 AM

There were 400 extra deaths [from the flu].

2:31

On average over the past ten years  we observed 2900 extra deaths per year in the Netherlands

2:37
Which makes the flu a really serious infectious disease.

2:40

If you consider all infectious diseases, influenza is the most common and the most deadly.

***Secretary of state Paul Blokhuis:***

2:46

The flu vaccine has been thoroughly reviewed and we are very transparent about what's in there,

2:53

also about any side effects.

***Prof. Dr. Eric van Gorp:***

2:56

The flu vaccine is effective and safe, because vaccines that come on the market in the Netherlands

3:01 AM

have been extensively tested, in particular for safety. But of course also on effectiveness,

3:06

so you can assume that Influenza vaccinations are safe

***Dr. Ted van Essen:***

3:08

[Side effects] are kept by LAREB in the Netherlands. There you can report side effects,

03:15

and based on this feedback an annual report is issued.

3:17

All you will notice from the flu shot is that you get some pain in the arm where you got it.

3:20

But that's not a side effect, that's where the vaccine starts to work.

***Prof. Erik Scherder:***

3:25

In addition to taking the vaccines, people can take care of

3:29

having an active lifestyle. That actually means they sleep well,

3:33

and also exercise enough during the day.

3:36

The two are closely related. By exercising and sleeping well, you also strengthen your immune system.

***Dr. Ted van Essen:***

3:41

The myth that you can get autism from vaccinations has been around for years. That's an absolute hoax.

3:47

A doctor in the United Kingdom, who reported this misinformation, has been convicted for spreading   this myth to the world,

3:53

as after investigation [his claims] turned out to be absolutely fraudulent.

3:58

There is no indication whatsoever that you can get autism from vaccinations.

4:03

People just keep spreading this myth

4:06

and it is very difficult to block such messages,

4:10

but it really is time to get this kind of nonsense out of the world.

***Prof. Dr. Eric van Gorp:***

4:12

A vaccine is considered unsafe if there are [serious] side effects.

4:16

A vaccine in general can cause side effects, mild side effects because your immune system is activated.

4:22

These are expected effects and side effects.

4:26

If unexpected or serious side effects occur,

4:28

it means the end of that vaccine.

***Dr. Ted van Essen:***

4:33

A vaccine contains small pieces of the virus that are dead.

4:39

That's why, if you get the vaccination you cannot get the flu.

4:43

Because the virus in it no longer works.

4:49

The body will make antibodies against those small pieces of the virus.

4:53
If you encounter the actual flu, the body recognizes the virus immediately, and starts using antibodies [produced due to the vaccine] to fight it.

5:01

Thus you prevent serious disease.

***Secretary of state Paul Blokhuis:***

5:03

Don't believe all the nonsense you read on the internet. Consult the RIVM (= Dutch CDC) website,

5:08

it contains valid information.

5:10

Also about what exactly is in [the vaccine], we really have nothing to hide.

5:15

Fear is totally unfounded when it comes to an influenza vaccination.

***Prof. Dr. Leonard Hofstra:***

05:17

There are still people who believe the Earth is flat, and also people who think that

05:23

the immune system is weakened when you get vaccinated, this is absolute nonsense.

05:29

The vaccine is only given for a specific virus for which you are being vaccinated.

05:35

It makes your immune system much stronger in fighting this specific [virus].

05:38

The rest of the system is completely unaffected by this.

***Secretary of state Paul Blokhuis:***

05:42

I would say to the people who get an invitation from their GP to get the flu shot, that the GPs are doing their very best to get people vaccinated,

05:47

make use of it, because it is completely safe.

05:51

I can imagine people are scared.

05:53

That they might think “will it be crowded while I am waiting for a flu shot?”, but this is absolutely not the case.

6:00

The GPs have organized it well, so no fear while getting the vaccine.

***Prof. Erik Scherder:***

6:06

I also take a flu vaccine myself, because I want to protect myself against the effects of the flu,

6:10 AM

and i also want to protect others, by making the chance of getting sick as small as possible.

***Prof. Dr. Leonard Hofstra:***

06:15

so get vaccinated.

6:16 AM

You do this not only for yourself, but also for your loved ones.
