# Supplementary material for: A media intervention applying debunking versus non-debunking content to combat vaccine misinformation in elderly in the Netherlands: A digital randomised trial
Source: eClinicalMedicine. 2021 May 15;35:100881. doi: 10.1016/j.eclinm.2021.100881 (PMC8176124; doi:10.1016/j.eclinm.2021.100881)
Supplement: Supplementary file 3 [file mmc3.docx]

**Captions for supplementary material**


File 1: **Study survey**
The diagnostic survey developed for the study, containing questions on (A) demographic information (11 questions), (B) governmental trust on influenza vaccination (7 questions), (C) vaccine hesitancy (10 questions), and (D) myths and knowledge about influenza and COVID-19 (6 questions)

File 2: **Time-stamped transcripts of the interventional videos**The transcript sections are color-coded on being related to Debunking fragments (labelled yellow, only in Video 2 (Debunking)), Social Norm Modelling (labelled pink), and Information (labelled green) in the respective contents of either video.
